# Polymorphisms in Adipokines in Mexican Children with Obesity

**DOI:** 10.1155/2019/4764751

**Published:** 2019-07-01

**Authors:** Angélica Saraí Jiménez-Osorio, Alma Olivia Aguilar-Lucio, Helios Cárdenas-Hernández, Claudette Musalem-Younes, Jacqueline Solares-Tlapechco, Paula Costa-Urrutia, Oscar Medina-Contreras, Julio Granados, Martha Eunice Rodríguez-Arellano

**Affiliations:** ^1^Laboratorio de Medicina Genómica del Hospital Regional Lic, Adolfo López Mateos, ISSSTE, Av. Universidad 1321, Florida, C.P. 01030, Álvaro Obregón, Mexico City, Mexico; ^2^Servicio de Neonatología del Hospital Regional Lic, Adolfo López Mateos, ISSSTE, Av. Universidad 1321, Florida, C.P. 01030, Álvaro Obregón, Mexico City, Mexico; ^3^Laboratorio de Investigación en Inmunología y Proteómica, Hospital Infantil de México Federico Gómez, Mexico City, Mexico; ^4^División de Inmunogenética, Departamento de Trasplantes, Instituto Nacional de Ciencias Médicas y Nutrición Salvador Zubirán, C.P. 14080, Mexico City, Mexico

## Abstract

The high prevalence of childhood obesity in Mexico is alarming in the health-science field. We propose to investigate the contribution of adipokines and cytokines polymorphisms and common BMI/obesity-associated loci, revealed in genome-wide association studies in Caucasian adult cohorts, with childhood obesity. This study included 773 Mexican-Mestizo children (5-15 years old) in a case-control study. The polymorphisms included were* ADIPOQ* (rs6444174),* TNF-α* (rs1800750),* IL-1β* (rs1143643),* IL-6* (rs1524107; rs2069845),* NEGR1 *(rs34305371),* SEC16B-RASAL2* (rs10913469),* TMEM18 *(rs6548238; rs7561317),* GNPDA2* (rs16857402),* LEP* (rs2167270),* MTCH2* (rs10838738),* LGR4-LIN7C-BDNF* (rs925946),* BCDIN3D-FAIM2* (rs7138803),* FTO* (rs62033400),* MC4R *(rs11872992),* MC4R* (rs17782313), and* KCTD15* (rs29942). No significant contribution was found with adipokines and cytokines polymorphisms in this study. Only both TMEM18 (rs6548238; rs7561317) polymorphisms were found associated with obesity (OR=0.5, P=0.008) and were in linkage disequilibrium (r^2^=0.87). The linear regression showed that the rs7561317 polymorphism of TMEM18 is negatively associated with obesity. This report highlights the influence of TMEM18 in Mexican-Mestizo children obesity, while adipokine and cytokine polymorphisms were not associated with it.

## 1. Introduction

Mexico has the highest prevalence of childhood obesity worldwide [1]. Children obesity is a predictive factor for adult obesity [2] and leads to the development of insulin resistance, diabetes, hypertension, cardiovascular disease, or cancer [3].

Children obesity develops in response to several factors, where nutrition and energy expenditure play a determinant role [4]. Adipose tissue is an endocrine organ that secretes hormones (adipokines) and proinflammatory cytokines [5]. Adiponectin is secreted by adipocytes with antidiabetic, antiatherogenic, and anti-inflammatory functions [6]. During obesity adiponectin decreases and serum levels are negatively correlated with body mass index (BMI) in prepubertal children [7]. An increase in proinflammatory cytokines like Tumor Necrosis Factor-alpha (TNF-*α*), interleukin-1 beta (IL-1*β*), and interleukin-6 (IL-6) suppresses adiponectin expression [8], which is regulated by the* ADIPOQ* gene, and single nucleotide polymorphisms (SNPs) in this gene have been associated with obesity-related traits [9].

Also, genetic factors also have a significant contribution to obesity as evidenced by the large disparities observed between ethnic groups. Results of large cohort studies showed that American Indian, Mexican, and Hispanic children have a higher risk to develop obesity than Africans children [10]. It has been estimated that obesity heritability goes from 6% to 85% among several populations. Genome Wide Association Studies (GWAS) have revealed several loci highly associated with body mass index (BMI), waist circumference, and body fat percentage (BF%) among populations [11–13]. Fat mass and obesity-associated gene (*FTO*) has been widely linked to obesity among populations, and posterior replications have shown an additional signal in the melanocortin 4 receptor (*MC4R*) locus. From the Genetic Investigation of Anthropometric Traits (GIANT) consortium, additional loci were associated with obesity, like transmembrane protein 18 (*TMEM18*), potassium channel tetramerization domain containing 15 (*KCTD15*), glucosamine-6-phosphate deaminase 2 (*GNPDA2*), Src homology 2B (*SH2B*) family member 1 (*SH2B1*), mitochondrial carrier 2 (*MTCH2*), the melanocortin 4 receptor (*MC4R*), and the neuronal growth regulator 1 (*NEGR1*) genes [14]. Thus, the aim of this study was to evaluate the association between polymorphisms previously associated in GWAS in Caucasian adult population, adipokines, and proinflammatory cytokines with obesity in Mexican-Mestizo children.

## 2. Methodology

### 2.1. Study Design

A case of control study was conducted in 773 children ranging from 5 to 15 years old, from the children obesity cohort COIPIS (Cohorte de Obesidad Infantil-Proyecto Infancia Saludable) from Hospital Regional Lic, Adolfo López Mateos, ISSSTE (Instituto de Seguridad y Servicios Sociales de los Trabajadores del Estado) [15].

After parents signed an informed consent to include their children in this study, anthropometric measurements (weight, height, BMI, and BF%) were determined. BMI was calculated as weight (kg) divided by height (m) squared. Obesity was defined according to the World Health Organization (WHO) Child Growth Standard for children as BMI-for-age value over +2 SD [16] using the 95 percentile. The BF% was evaluated using a Body composition analyzer with an accurate stadiometer (InBody J10). A whole blood sample was taken to evaluate clinical parameters at fasting: glucose, glycated hemoglobin (HbA1c), creatinine, triglycerides (TAG), total cholesterol (CHOL), and high-density lipoprotein cholesterol (HDL).

An exclusion criterion was related to children with foreign parents and grandparents. This study was conducted in accordance with the Declaration of Helsinki and approved by the Hospital Regional Lic, Adolfo López Mateos Research, Ethics, and Biosafety Committees (registration number 245.2012).

### 2.2. DNA Extraction and Genotyping

Genomic DNA was obtained from 200 *μ*l of the whole blood-EDTA with the InviMag Blood DNA Mini Kit, using an automated system (InviGenius, Stratec). For genotyping, we selected 18 SNPs (Table 1) which were amplified using predesigned 5′ exonuclease TaqMan genotyping assays on a 7500 series Real-Time PCR system (Applied Biosystems, Foster City, CA, USA).

## 3. Statistical Analysis

The results of continue variables are presented as mean ± standard deviation for Gaussian distribution and analyzed by the Student t test. For non-Gaussian distribution, the continuous variables are presented as median and interquartile range and analyzed by Mann-Whitney test. A chi-squared test was performed to estimate the Hardy-Weinberg equilibrium in controls. Pearson correlations (R) were used to assess linkage disequilibrium (LD) between SNPs in* TMEM18*,* IL-6*, and* MC4R* genes using Haploview® Software (Broad Institute, Cambridge, MA, USA). Statistical power of a case-control outcome design was carried out including 17% of childhood obesity prevalence [17], gene only and allele frequencies from 0.02 to 0.5. To evaluate the effect of the SNPs on children obesity, a logistic regression was used, including dominant and additive inheritance models. In addition, for further assessment a linear model was used to test the significant loci (resulted from logistic regression) on BMI, BM%, and clinical parameters. All statistical analyses were adjusted by gender, age, and BMI (in the case of logistic regression) and were performed using STATA12 (StataCorp, Texas). The statistical power calculation was performed using Quanto® Software (USC Biostats, California, USA). Data (.dta) used to support the findings of this study are available from the corresponding author upon request.

## 4. Results

Clinical and anthropometrical characteristics are summarized in Table 2. This study included 204 children with obesity and 569 children without obesity. BF%, glucose, creatinine, TAG, and CHOL levels were found higher in children with obesity. However, the levels of these clinical outcomes in the obesity group fall in normal ranges. Only HDL levels decreased in this group. There was no difference in HbA1c concentration between groups.

All the SNPs analyzed were in Hardy-Weinberg equilibrium. The allelic distribution of the SNPs is shown in Table 3. Only rs6548238 and rs7561317 SNPs in* TMEM18* were associated in the dominant and additive inheritance models. Both polymorphisms confer significant protection against obesity. These polymorphisms were in linkage disequilibrium in our studied population (r^2^=0.87).

A marginal association was found between rs925946 in* LGR4-LIN7C-BDNF* and rs1800750 in* TNF-α* with obesity but does not reach statistical significance. In this study we did not identify any association between* ADIPOQ* or any cytokine polymorphism with obesity (Table 3).

To evaluate the effects of the associated polymorphisms on BMI and BF%, a linear regression was performed polymorphisms (Table 4). Both* TMEM18* rs6548238 and rs7561317 loci were only associated BF% after adjustment by age and gender. However, the rs7561317 polymorphism was significantly associated with BMI. There was no association between these polymorphisms and clinical parameters.

## 5. Discussion

It is well recognized that adipokines and cytokines homeostasis plays an important role in obesity condition. Additionally, GWAS in Caucasian populations revealed many common loci associated with adult obesity [18]. Here, we analyzed the effect of several of these polymorphisms, as well as adipokines (*ADIPOQ*) and cytokines (*TNF*-*α*,* IL-1β*, and* IL-6*).

We observed a significant association between* TMEM18 * (rs7561317) and children obesity; however, we did not observe significant association between adipokines and obesity in our study. A meta-analysis of the influence of adipokine polymorphisms in adipokine genes (*LEP, ADIPOQ, IL-1β, IL-6*, and* TNF-α*) in obesity susceptibility showed that there was not association with the risk of obesity and LEP variants in adults. Interestingly, the results of this meta-analysis suggested that polymorphisms in the adipokine genes,* ADIPOQ, IL-1β, IL-6, and TNF-α*, increase the risk of obesity [19]. Our results are consistent with three studies in Mexican children that showed no contribution of several polymorphisms in* ADIPOQ* gene (rs2241766, rs182052, rs266729, and rs822393) in children obesity [9, 20, 21]. A similar effect was observed in* TNF-α* and interleukin-10 [22], suggesting that genetic variants in adipokines do not have a significant effect in Mexican-Mestizo children obesity.

Polymorphisms in* FTO* and* MC4R* have been associated with obesity in children. A recent meta-analysis which included 13 articles published between 2011 and 2015, comprising 15,613 participants, shows that the polymorphism rs9939609 in* FTO *was significantly associated with an increased risk of obesity in children and adolescents in several populations, except Mexicans [23]. Another recent report exposed that the rs9939609 polymorphism interacts with the Native American-origin polymorphism rs9282541, in the ATP-binding cassette transporter (*ABCA1*). Also, in the presence of the rs9282541 risk allele, the rs9939609 variant was not associated with BMI in Mexican adults, but this interaction was not observed in children [24]. This lack of significance was attributed to the to sample size; however the study of Mejía-Benitez (REF) showed no association between several polymorphisms in* FTO* with obesity in Mexican children, using a larger sample size. We propose that large cohorts during extensive periods could help to understand the real contribution of* FTO *variants in Mexican children.

The* TMEM18* rs7561317 variant has been widely studied. This polymorphism, together with rs10913469 in* SEC16B* gene, was associated with adult obesity in a Japanese population [25] and Caucasians children [26]. On the contrary, the minor allele of* TMEM18 *rs7561317 was related to underweight in Czech adolescents [27]. This polymorphism has also been associated with BMI in Mexican children [28]. In our study, after adjustment by age, sex, and BMI, we observed the same association. Thus, this report indicates that* TMEM18* rs7561317 has an effect on obesity development at all stages of life development.

## 6. Conclusions

Here we report no association of adipokines and cytokines polymorphisms with children obesity. However, the rs7561317 polymorphism in* TMEM18* gene is associated with BMI and BF% in our sample of Mexican-Mestizo children.

## Figures and Tables

**Table 1:** Single nucleotide polymorphisms evaluated in this study.

SNP	Gene	Chromosome
rs34305371	*NEGR1*	1
rs10913469	*SEC16B-RASAL2*	1
rs6548238	*TMEM18*	2
rs7561317	*TMEM18*	2
rs1143643	*IL-1beta*	2
rs6444174	*ADIPOQ*	3
rs16857402	*GNPDA2*	4
rs1800750	*TNF-alpha*	5
rs1524107	*IL-6*	7
rs2069845	*IL-6*	7
rs2167270	*LEP*	7
rs10838738	*MTCH2*	11
rs925946	*LGR4-LIN7C-BDNF*	11
rs7138803	*BCDIN3D-FAIM2*	12
rs62033400	*FTO*	16
rs11872992	*MC4R*	18
rs17782313	*MC4R*	18
rs29942	*KCTD15*	19

**Table 2:** Clinical and anthropometrical measurements according to obesity status.

	Nonobesity	Obesity	*P*
n	569	204	
Age	9.3 (6.1–12.9)	11.3 (9.2–13.4)	<0.001
Male n, (%)	275 (48.3)	120 (58.8)	0.032
Body Fat (%)	23.9 ± 10.2	38.27 ± 8.7	<0.001
BMI	17.3 (15.7–19.7)	24.98 (22.5–21.6)	<0.001
Glucose	96 (90–103)	98 (92–105)	0.0199
HbA1c	5.4 (5.1–5.6)	5.44 (5.2–5.7)	0.2182
Creatinine	0.91 (0.77–1.07)	0.98 (0.83–1.14)	0.0018
TAG	87.5 (66.5–113)	120 (90–167)	<0.001
CHOL	162 (142–180)	170 (152–191)	0.0023
HDL	61.34 ± 12.34	56.77 ± 12.05	<0.001

Data are presented as mean ± SD for continuous variables with normal distribution, median (interquartile range) for continuous variables with nonnormal distribution. BMI: body mass index (kg/m^2^), HbA1c: glycated hemoglobin, TAG: triglycerides, CHOL: cholesterol, and HDL: high density lipoprotein cholesterol.

**Table 3:** Logistic regression of polymorphisms studied.

SNP	Gene	MAF	Allele	OR (95% CI), P_*dom*_	OR (95% CI), P_*add*_
rs34305371	*NEGR1*	0.02	G	0.92, (0.4-1.97), 0.841	0.88, (0.4-1.8), 0.746
rs10913469	*SEC16B-RASAL2*	0.02	C	1, (0.7-1.36), 0.902	1, (0.8-1.3), 0.826
rs6548238	*TMEM18*	0.05	T	0.5, (0.3-0.83), 0.008	0.53, (0.3-9), 0.013
rs7561317	*TMEM18*	0.06	A	0.52, (0.3-0.8), 0.008	0.5, (0.3-0.9), 0.012
rs1143643	*IL-1beta*	0.25	T	0.93, (0.7-1.3), 0.711	0.88, (0.7-1.16), 0.372
rs6444174	*ADIPOQ*	0.03	C	0.65, (0.3-1.3), 0.224	0.63, (0.3-1.2), 0.193
rs16857402	*GNPDA2*	0.12	C	1.06, (0.7-1.6), 0.743	1.06, (0.7-1.5), 0.745
rs1800750	*TNF-alpha*	0.04	A	1.8, (0.9-3.6), 0.068	1.75, (0.9-3.3), 0.087
rs1524107	*IL-6*	0.47	T	0.96, (0.7-1.3), 0.847	0.94, (0.7-1.2), 0.670
rs2069845	*IL-6*	0.2	G	1.1, (0.8-1.5), 0.551	1.08, (0.8-1.44), 0.606
rs2167270	*LEP*	0.49	G	0.98, (0.7-1.4), 0.916	0.99, (0.8-1.25), 0.990
rs10838738	*MTCH2*	0.39	G	1.3, (0.9-1.8), 0.126	1.1, (0.8-1.4), 0.379
rs925946	*LGR4-LIN7C-BDNF*	0.14	T	0.7, (0.5-1.03), 0.072	0.78, (0.6-1.1), 0.128
rs7138803	*BCDIN3D-FAIM2*	0.23	A	1.03, (0.7-1.45), 0.825	1.04, (0.8-1.36), 0.775
rs62033400	*FTO*	0.28	G	1.02, (0.7-1.5), 0.873	1.02, (0.7-1.3), 0.880
rs11872992	*MC4R*	0.24	A	1.2 (0.8-1.8), 0.290	1.2, (0.85-1.7), 0.276
rs17782313	*MC4R*	0.08	C	0.8, (0.5-1.3), 0.454	1.02, (0.6-1.6), 0.912
rs29942	*KCTD15*	0.42	A	0.77, (0.5-1.1), 0.158	0.9, (0.7-1.14), 0.402

OR adjusted by age, sex, and BMI. Inheritance models = dom: dominant; add: additive. MAF: minor allele frequency.

**Table 4:** Linear regression of BMI and body fat percentage including *TMEM18* polymorphisms as additive model.

SNP	Gene	*β* BMI (95% CI)	*P*	*β* Body Fat (95% CI)	*P*
rs6548238	*TMEM18*	-1.15 (-2.4 – 0.12)	0.075	-0.025 (-0.04 – -0.004)	0.018
rs7561317	*SFRS10-ETV-DGKG*	-1.23 (-2.4 – -0.02)	0.046	-0.03 (-0.05 – -0.01)	0.001

## Data Availability

Data (.dta) used to support the findings of this study are available from the corresponding author upon request.

## References

[1] ENSANUT Encuesta Nacional de Nutrición. https://ensanut.insp.mx/ensanut2016/descarga_bases.php.

[2] Seyednasrollah F., Mäkelä J., Pitkänen N. (2017). Prediction of adulthood obesity using genetic and childhood clinical risk factors in the cardiovascular risk in young finns study. *Circulation: Cardiovascular Genetics*.

[3] Llewellyn A., Simmonds M., Owen C. G., Woolacott N. (2016). Childhood obesity as a predictor of morbidity in adulthood: a systematic review and meta-analysis. *Obesity Reviews*.

[4] Dev D. A., McBride B. A., Fiese B. H. (2013). Risk factors for overweight/obesity in preschool children: an ecological approach. *Childhood Obesity*.

[5] Ronti T., Lupattelli G., Mannarino E. (2006). The endocrine function of adipose tissue: an update. *Clinical Endocrinology*.

[6] Achari A. E., Jain S. K. (2017). Adiponectin, a therapeutic target for obesity, diabetes, and endothelial dysfunction. *International Journal of Molecular Sciences*.

[7] Madeira I. R., Carvalho C. N., Gazolla F. M., Pinto L. W., Borges M. A., Bordallo M. A. (2009). Impact of obesity on metabolic syndrome components and adipokines in prepubertal children. *Jornal de Pediatria*.

[8] Berg A. H., Scherer P. E. (2005). Adipose tissue, inflammation, and cardiovascular disease. *Circulation Research*.

[9] Muñoz-Yáñez C., Pérez-Morales R., Moreno-Macías H. (2016). Polymorphisms FTO rs9939609, PPARG rs1801282 and ADIPOQ rs4632532 and rs182052 but not lifestyle are associated with obesity related-traits in Mexican children. *Genetics and Molecular Biology*.

[10] Ferdinand K. C., Nasser S. A. (2015). Racial/ethnic disparities in prevalence and care of patients with type 2 diabetes mellitus. *Current Medical Research and Opinion*.

[11] Andreasen C. H., Andersen G. (2009). Gene–environment interactions and obesity—Further aspects of genomewide association studies. *Nutrition Journal *.

[12] Zillikens M. C., Demissie S., Hsu Y.-H. (2017). Large meta-analysis of genome-wide association studies identifies five loci for lean body mass. *Nature Communications*.

[13] Rask-Andersen M., Karlsson T., Ek W. E., Johansson Å. (2019). Genome-wide association study of body fat distribution identifies adiposity loci and sex-specific genetic effects. *Nature Communications*.

[14] Goodarzi M. O. (2018). Genetics of obesity: what genetic association studies have taught us about the biology of obesity and its complications. *The Lancet Diabetes & Endocrinology*.

[15] Costa-Urrutia P., Vizuet-Gámez A., Ramirez-Alcántara M. (2019). Obesity measured as percent body fat, relationship with body mass index, and percentile curves for Mexican pediatric population. *PLoS ONE*.

[16] de Onis M. (2008). The who child growth standards. *Pediatric Nutrition in Practice*.

[17] Hernández-Cordero S., Cuevas-Nasu L., Morán-Ruán M. C., Méndez-Gómez Humarán I., Ávila-Arcos M. A., Rivera-Dommarco J. A. (2017). Overweight and obesity in Mexican children and adolescents during the last 25 years. *Nutrition & Diabetes*.

[18] Willer C. J., Speliotes E. K., Loos R. J. (2009). Six new loci associated with body mass index highlight a neuronal influence on body weight regulation. *Nature Genetics*.

[19] Yu Z., Han S., Cao X., Zhu C., Wang X., Guo X. (2012). Genetic polymorphisms in adipokine genes and the risk of obesity: a systematic review and meta-analysis. *Obesity*.

[20] León-Mimila P., Villamil-Ramírez H., Villalobos-Comparán M. (2013). Contribution of common genetic variants to obesity and obesity-related traits in mexican children and adults. *PLoS ONE*.

[21] Peralta Romero J. D. J., Karam Araujo R., Burguete García A. I. (2015). ADIPOQ and ADIPOR2 gene polymorphisms: association with overweight/obesity in mexican children. *Boletín Médico del Hospital Infantil de México*.

[22] Vashi N., Stryjecki C., Peralta-Romero J. (2016). Genetic markers of inflammation may not contribute to metabolic traits in Mexican children. *PeerJ*.

[23] da Silva T. E., Andrade N. L., Cunha D. d., Leão-Cordeiro J. A., Vilanova-Costa C. A., Silva A. M. (2018). The FTO rs9939609 polymorphism and obesity risk in teens: Evidence-based meta-analysis. *Obesity Research & Clinical Practice*.

[24] Villalobos-Comparán M., Antuna-Puente B., Villarreal-Molina M. T. (2017). Interaction between FTO rs9939609 and the Native American-origin ABCA1 rs9282541 affects BMI in the admixed Mexican population. *BMC Medical Genetics*.

[25] Hotta K., Nakamura M., Nakamura T. (2009). Association between obesity and polymorphisms in SEC16B, TMEM18, GNPDA2, BDNF, FAIM2 and MC4R in a Japanese population. *Journal of Human Genetics*.

[26] Namjou B., Keddache M., Marsolo K. (2013). EMR-linked GWAS study: investigation of variation landscape of loci for body mass index in children. *Frontiers in Genetics*.

[27] Dušátková L., Zamrazilová H., Sedláčková B. (2013). Association of obesity susceptibility gene variants with metabolic syndrome and related traits in 1,443 Czech adolescents. *Folia Biologica*.

[28] Abadi A., Peralta-Romero J., Suarez F. (2016). Assessing the effects of 35 European-derived BMI-associated SNPs in Mexican children. *Obesity*.

